# Simultaneous Carotid Artery Stenting and Coronary Artery Bypass Grafting in Urgent Patients: A Single Center Experience

**DOI:** 10.3390/jcm13237180

**Published:** 2024-11-26

**Authors:** Mariafrancesca Fiorentino, Elisa Mikus, Roberto Nerla, Diego Sangiorgi, Andrea Ruggiero, Alberto Tripodi, Fausto Castriota, Carlo Savini

**Affiliations:** 1 Cardiovascular Department, Maria Cecilia Hospital, GVM Care & Research, 48033 Cotignola, Italy; elisamikus@yahoo.it (E.M.); robertonerla83@gmail.com (R.N.); dsangiorgi@gvmnet.it (D.S.); atripodi@gvmnet.it (A.T.); fcastriota@msn.com (F.C.); csavini@gvmnet.it (C.S.); 2 Department of Experimental Diagnostic and Surgical Medicine (DIMEC), University of Bologna, 40126 Bologna, Italy; andrea.ruggiero5@studio.unibo.it

**Keywords:** coronary artery bypass grafting, carotid artery stenting, stroke, postoperative bleeding

## Abstract

**Background**: Coexisting coronary artery disease and critical carotid stenosis present challenges in revascularization, particularly in urgent cases requiring surgery. Combining carotid artery stenting (CAS) with coronary artery bypass grafting (CABG) has gained popularity. **Methods**: This study analyzed 36 patients who underwent simultaneous CAS and CABG from 2014 to 2024. CAS was performed first, with the patient awake for real-time neurocognitive assessment. A clopidogrel loading dose was administered three hours post-surgery. From postoperative day 1, dual antiplatelet therapy was initiated. **Results**: The median age was 72 years (64–77) and 22.2% were females. The median EuroSCORE II was 2.80 (2.06–3.58). Nine patients (25%) underwent associated procedures. The median cardiopulmonary bypass and cross-clamp times were 66 (54–89) and 51 (41–72) minutes. We observed no in-hospital mortality and no postoperative stroke. The median postoperative bleeding in 24 h was 500 mL and only one (2.8%) patient needed resternotomy for bleeding. The median ventilation time was 9 h (6–12). The median intensive care unit and postoperative length of stay were 2 (2–4) days and 8 (7–11) days, respectively. The median follow-up time was 6 years. Survival at 1, 5, and 10 years was 93.7%, 81.5%, and 60.2%, respectively, while freedom from PTCA/PCI at 1, 5, and 10 years was 100%, 96.7%, and 87.5%, respectively. **Conclusions**: Simultaneous CAS and CABG is a safe and effective procedure with low in-hospital mortality and morbidity. Our protocol carries a low risk of perioperative stroke. Antiplatelet therapy administration on the day of surgery does not increase the risk of postoperative bleeding.

## 1. Introduction

Concomitant coronary artery disease (CAD) requiring surgery and critical carotid stenosis frequently coexist due to their common atherosclerotic etiology [1,2]. A direct association between the severity of CAD and the presence of carotid artery stenosis has been documented in patients undergoing coronary angiography highlighting the bidirectional nature of these vascular pathologies [3]. Recently, the incidence of carotid stenosis in patients with ischemic heart disease scheduled for surgical revascularization has been estimated to be approximately 13% [4]. On the other hand, the prevalence of severe coronary artery disease in patients with bilateral critical carotid stenosis is even higher, reaching up to 65% in some studies [5]. Nevertheless, there is no current consensus on the optimal timing, sequence, or methods of revascularization, particularly in patients with urgent surgical indications for coronary artery disease. In fact, a combined surgical approach with simultaneous coronary artery bypass grafting (CABG) and carotid tromboendarterectomy (CEA) suffers from a significant risk of periprocedural stroke and death [6,7]. In a study involving 111 patients who underwent simultaneous CEA and CABG [6], the 30-day all-cause mortality rate was 6.3%. Postoperative neurological events occurred in 7.2% of patients, with 4.5% experiencing disabling strokes and 7.2% suffering transient ischemic attacks (TIA). These rates were notably higher than those reported for isolated coronary artery disease treatment. Conversely, a staged surgical approach involving CABG followed by CEA is associated with an elevated risk of stroke, while performing CEA prior to CABG increases the risk of perioperative myocardial infarction. This is clearly evident in a systematic review and meta-analysis [8], which found that carotid endarterectomy followed by staged coronary artery bypass grafting was associated with the lowest perioperative stroke and TIA rates. These rates were significantly lower compared to those observed with staged CABG and CEA. However, this approach was also linked to the highest perioperative mortality and myocardial infarction rates. Moreover, in most cases, coronary artery disease requires urgent intervention and cannot be deferred until carotid disease is addressed, making the sequencing of these interventions a critical concern. In this context, synchronous or staged carotid artery stenting (CAS) combined with CABG has emerged as a feasible and promising therapeutic strategy, demonstrating low morbidity and mortality rates in this high-risk patient cohort. In fact, in a 2019 study by Xiang et al. [9], 245 patients undergoing combined carotid and coronary revascularization were analyzed, of which 208 were treated with carotid stenting followed by staged coronary surgery. The outcomes of these patients were comparable to those of patients who underwent simultaneous combined carotid and coronary surgery. However, in their cohort, only five patients underwent a hybrid procedure involving both carotid stenting and coronary artery bypass grafting (CABG). The 2018 European Society of Cardiology (ESC) guidelines [10] provide guidance for the management of patients with coexisting CAD and carotid artery disease. They recommend that, in patients scheduled for CABG with a recent history of transient ischemic attack (TIA) or stroke, carotid revascularization should be considered (Class IIa) for those with 50–99% carotid stenosis, with CEA recommended as the first-line treatment. For neurologically asymptomatic patients, carotid revascularization may be considered (Class IIb) for those with bilateral 70–99% carotid stenosis or for those with 70–99% carotid stenosis accompanied by contralateral occlusion.

Given the complexities of managing concomitant CAD and critical carotid stenosis, we sought to review our experience with a combined approach to carotid and coronary artery disease in an urgent setting. Our aim is to evaluate and discuss the potential benefits and risks of this combined strategy, contributing to the growing body of evidence on the most effective revascularization protocols in this challenging patient population.

## 2. Materials and Methods

### 2.1. Study Population

All adult patients (age > 18 years old) who underwent urgent simultaneous CAS and CABG were included in the present study. All patients suffered from coronary artery disease with an indication to urgent cardiac surgery due to anatomical or clinical reasons and concomitant significant bilateral carotid artery disease (70%) as an incidental finding during pre-operative work-up. From a clinical perspective, we classified patients as urgent if they presented with acute coronary syndromes (NSTEMI or unstable angina). From an anatomical standpoint, urgency was defined by the presence of critical left main coronary artery stenosis or left main equivalent disease (critical stenosis of both the proximal LAD and the proximal LCx). In these patients, the intervention was typically performed within 7 days of diagnosis or during the same index hospitalization. The decision to limit our study to urgent cases stems from our belief that a combined procedure is justified only in patients who cannot delay cardiac surgery until after treatment of their carotid disease. No sample size calculation was performed, and all available patients were included in this study. Therefore, we retrospectively analyzed 36 patients undergoing the two procedures simultaneously from January 2014 to January 2024 in our institution. The study was conducted in accordance with the Declaration of Helsinki, and the protocol was approved by the Romagna Ethics Committee on 20 November 2019 (Prot. 9953/2019 I.5/93). Patients enrolled provided written consent for data collection as part of clinical routine practice, quality assessment and for scientific research purposes. We retrospectively analyzed preoperative, intra-operative and postoperative clinical data of all patients using medical records, and every possible effort was made to reduce missing information. Only complete cases were analyzed. The primary endpoints were in-hospital mortality, defined as any death occurring before discharge from the index hospitalization and the occurrence of any postoperative adverse event, with particular focus on bleeding and neurological complications. Additionally, patients’ follow-up consisted of either out-patient appointment or telephone consultation to assess medium and long-term survival and freedom from endovascular procedures.

### 2.2. Operative Strategy

All patients scheduled for coronary artery bypass surgery underwent preoperative carotid ultrasound as per recommended clinical guidelines [10]. When significant bilateral carotid artery stenosis (≥70%) or significant unilateral carotid stenosis with contralateral occlusion was detected on ultrasound, a confirmatory CT angiography was performed [11]. Additionally, a brain CT scan was routinely performed in these patients to identify any pre-existing ischemic lesions. In all patients, carotid artery stenting and coronary artery bypass grafting were performed concurrently in a hybrid operating room equipped with an angiography system. Carotid artery stenting was performed first, with the patient awake to enable real-time neurocognitive assessment, utilizing either XACT, Roadsaver, or Carotid Wallstent stents. Embolic protection was employed in all cases, using either the Mo.Ma Ultra proximal cerebral protection device or distal filters. The percutaneous transfemoral approach was used in the stenting procedure. Heparin was administered at a dose of 200 IU/kg to achieve an activated clotting time (ACT) of greater than 200 s. In all patients a post-dilatation after stent deployment was performed. Following the stenting procedure, patients were administered total intravenous anesthesia, and coronary artery bypass surgery was predominantly performed using the standard on-pump technique in 31 patients, with a small proportion of procedures (13.9%) conducted off-pump. The surgery was conducted under general heparinization targeting an ACT of greater than 480 s. The graft of choice for bypassing the left anterior descending artery was the internal mammary artery, while other grafts were selected based on the patient’s age, comorbidities, and surgeon’s preference. Standard ascending aorta cannulation and venous cannulation were performed, and cold blood cardioplegia was administered. Upon completion of the procedure and after weaning the patient from cardiopulmonary bypass, complete heparin reversal was achieved by infusing a full dose of protamine and an antifibrinolytic agent. A loading dose of clopidogrel (600 mg) was administered three hours post-surgery, in case of no major bleeding. Starting on postoperative day 1, dual antiplatelet therapy was initiated, consisting of 100 mg of cardioaspirin and 75 mg of clopidogrel, and continued for 1–3 months, according to the type of stenting used. The remaining postoperative management at our institution is consistent with the standard protocols for patients undergoing isolated coronary artery bypass surgery. In detail, once in the intensive care unit (ICU), patients are usually extubated on postoperative day (POD) 0, after evaluation of thoracic drains output, state of consciousness and gas exchanges. They usually spend their first night in the ICU and the second night in a high dependency unit (HDU) if the clinical conditions allow it. On POD 1, all patients are mobilized into an armchair. Thoracic drains are removed on POD 2, and patients are moved to the ward where they are fully mobilized. This usually allows us to discharge patients home on POD 7.

### 2.3. Statistical Analysis

After checking for normality using the Shapiro–Wilk test, continuous variables were reported as median and interquartile range (IQR); categorical variables were reported as absolute numbers and frequencies. Kaplan–Meier curves were used to evaluate long-term outcomes, and the percentages of surviving patients at 1, 5 and, 10 years were reported. All analysis was performed using STATA 17 SE (StataCorp LLC) and *p*-values < 0.05 were considered as statistically significant.

## 3. Results

### 3.1. Patients’ Characteristics

Patients’ characteristics are presented in Table 1. The median age of our population was 72 years, and eight patients (22.2%) were female. Comorbidities included hypertension (86.1%), diabetes mellitus (50%), dyslipidemia (63.9%), and current or previous smoking (50%). Additionally, patients had a median ejection fraction of 59%, a median creatinine level of 1.01 mg/dL and a median Euroscore II of 2.80, and only 11 patients (30.5%) were considered to be at high surgical risk (Euroscore II > 8%) [12]. Four patients (11.1%) had a history of preoperative stroke, and two (5.6%) experienced a transient ischemic attack (TIA). One patient (2.8%) underwent a redo operation, and another (2.8%) was supported by an intra-aortic balloon pump prior to surgery.

Regarding intraoperative characteristics (Table 2), the stents used were XACT in 38.9% of cases, Carotid Wallstent in 33.3%, and Roadsaver in 27.8%, and embolic protection was adopted in all cases, 44.4% with Mo.Ma Ultra proximal Cerebral Protection device and 65.6% with distal filters. Five procedures (13.9%) were performed off-pump; for the remaining cases, the median cardiopulmonary bypass (CPB) and cross-clamp times were 66 and 51 min, respectively. Median (IQR) grafts performed were 3 (2–3). Eight patients (22.2%) underwent associated surgical procedures, including both aortic valve replacement and mitral valve surgery.

### 3.2. In-Hospital Outcomes

In-hospital outcomes are summarized in Table 3. In our experience, we observed no in-hospital mortality and no postoperative strokes, with only one case (2.8%) of transient ischemic attack. Additionally, the median (IQR) postoperative bleeding within 24 h was 500 mL (325–700), and only one patient (2.8%) required resternotomy for bleeding, despite 26 patients (72.2%) needing red blood cell transfusions. Other postoperative adverse events observed included nine cases (25%) of postoperative atrial fibrillation, two patients (5.6%) who experienced postoperative myocardial infarction, and one patient (2.8%) requiring postoperative dialysis. Moreover, we report two (5.6%) cases of postoperative pneumonia, seven (19.4%) patients who required prolonged intubation and no wound complications. Median (IQR) ventilation time was 9 h (6–12), the median (IQR) intensive care unit stay, and median in-hospital stay were 2 (2–4) and 8 (7–11) days, respectively.

### 3.3. Follow-Up Results

**Figure 1:**
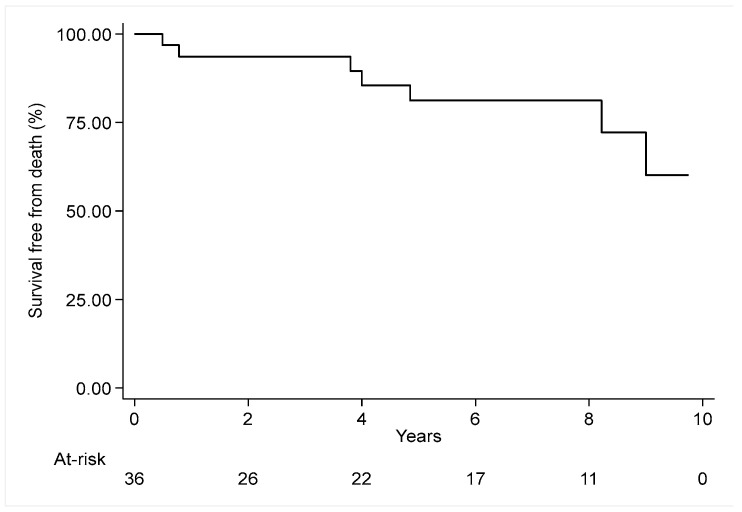
Kaplan–Meier curve for follow-up survival.

Patients’ follow-up consisted of either out-patient appointment or telephone consultation. We assessed survival and freedom from endovascular procedures including both percutaneous transluminal coronary angioplasty (PTCA) and carotid percutaneous transluminal angioplasty (PTA). The median follow-up time was 6 years. Survival rates at 1, 5, and 10 years was 93.7%, 81.5% and 60.2%, respectively (see Figure 1).

**Figure 2:**
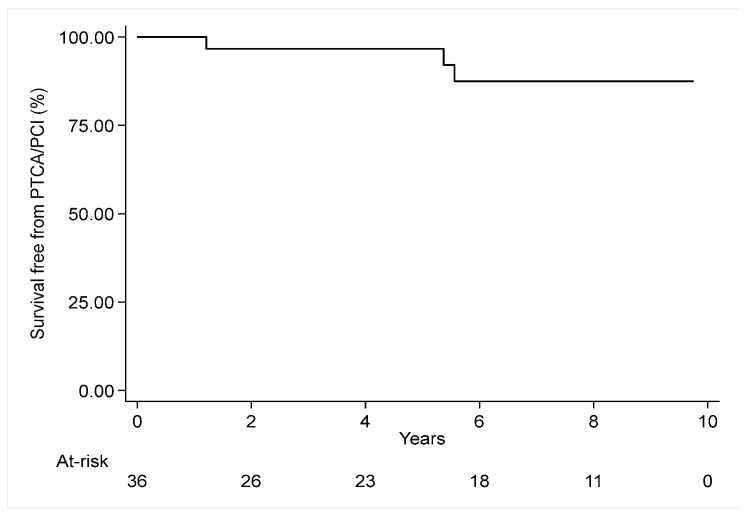
Kaplan–Meier curve for freedom from endovascular procedures.

During the follow-up period, three endovascular procedures were observed. These included one carotid artery stenting of the contralateral carotid artery and two percutaneous transluminal coronary angioplasties (PTCA). In all cases, disease progression was noted, affecting vessels that were not critically ill at the time of the initial surgery. Freedom from repeated endovascular procedures at 1, 5, and 10 years was 100%, 96.7%, and 87.5%, respectively (see Figure 2).

## 4. Discussion

In this retrospective study, we presented our limited experience with combined carotid artery stenting and coronary artery bypass grafting in a cohort of patients scheduled for urgent cardiac surgery. Despite the frequent coexistence of these two conditions [1,2,3,4,5], there is no consensus in the literature regarding either the revascularization techniques or the timing of interventions. Historically, carotid artery disease in patients with concomitant coronary artery disease has been treated surgically through traditional carotid endarterectomy (CEA), as also outlined in the most recent guidelines [10]. However, there remains no consensus on the optimal timing of the two procedures. The literature is, in fact, replete with studies comparing synchronous versus staged intervention. For example, in a study by Gopaldas et al. [13], 6153 patients who underwent CEA before or after CABG during the same hospital admission, but not on the same day, were compared to 16,639 patients who underwent both procedures on the same day. The authors found no significant difference in mortality or neurologic complications between the two approaches. However, staged procedures were associated with a higher overall complication rate and increased hospital costs compared to synchronous procedures. Conversely, a more recent meta-analysis [14] that included eleven studies and a total of 44,895 patients yielded different findings. Specifically, the synchronous CEA and CABG group exhibited a statistically significant reduction in the risk of myocardial infarction, along with an increased risk of stroke and mortality. However, other adverse events, such as transient ischemic attacks, postoperative bleeding, and pulmonary complications, were similar between the two groups. In summary, the concurrent performance of the two procedures may be associated with a higher risk of surgical mortality and stroke, while the staged execution of the interventions carries an increased risk of myocardial infarction. However, today, for the treatment of isolated symptomatic or asymptomatic carotid stenosis, surgical endarterectomy is not the only available option. In fact, randomized controlled trials [15,16] have demonstrated that CAS provides outcomes comparable to traditional surgery in terms of mortality and stroke risk, both in the short and long term. Consequently, carotid stenting is increasingly being utilized in patients with combined carotid and coronary artery disease. In comparison to simultaneous carotid endarterectomy, carotid stenting demonstrated no significant difference in a composite endpoint of postoperative myocardial infarction, neurological events, and mortality, in patients with significant carotid artery stenosis undergoing coronary bypass surgery [17]. Some authors [18,19] successfully advocate for performing carotid stenting up to six weeks prior to aortic coronary bypass surgery. This approach, though, would be impractical in our population which comprises patients indicated for urgent coronary surgery that must be conducted within approximately seven days of diagnosis or prior to discharge to home. We described a synchronous approach in which carotid artery stenting and coronary artery bypass grafting were performed concurrently in a hybrid operating room. We believe that the strength of our protocol lies in the implementation of real-time neurocognitive assessment. Specifically, this is achieved by performing carotid artery stenting while the patient is awake, allowing for immediate and continuous evaluation of neurological function throughout the procedure. This approach distinguishes our protocol from that proposed other studies [17], where a different methodology was utilized. By keeping the patient awake, we are able to detect any signs of cerebral ischemia or neurological compromise in real time. This real-time feedback provides an added layer of safety and precision, and we believe that it represents a significant advancement in the management of patients requiring simultaneous carotid and coronary interventions. In fact, we were able to maintain a very low incidence of neurological complications in our patient population and we reported 0% postoperative stroke and just one (2.8%) postoperative TIA. Nevertheless, in the event of a stroke, it would be promptly recognized, enabling us to halt the planned aorto-coronary bypass procedure and initiate the stroke management protocol that involves the immediate performance of a brain CT scan to confirm the diagnosis and guide subsequent treatment, such as thrombolysis or other appropriate interventions. Of course, since we did not perform postoperative imaging, we cannot exclude the possibility that small lesions may have occurred without clinical manifestations. However, it is important to note that all of these patients were fully anticoagulated following the carotid stenting procedure, which could have potentially contributed to hemorrhagic progression, even in the case of initially silent small lesions. Additionally, for the purposes of this study, we focused primarily on clinical neurological outcomes, which allowed us to assess the immediate and observable effects of the procedure. However, we acknowledge that the absence of imaging may have led to an underestimation of potential minor neurological events that did not present with clinical symptoms. Future studies with imaging follow-up would help to more comprehensively evaluate the safety and impact of our approach. Our results are similar to those of a recent study [20] analyzing the same technique, where carotid stenting was also performed with the patient awake, reporting postoperative incidences of stroke and TIA at 1.4% and 5.8%, respectively. By contrast, studies reporting an increased risk of stroke in patients undergoing simultaneous CAS and CABG [19] favored performing both procedures under general anesthesia. Regarding the management of anticoagulant and antiplatelet therapy in these patients, our approach involves administering heparin to achieve an activated clotting time (ACT) of greater than 200 s during the endovascular procedure. The remaining heparin dose is then administered to achieve an ACT of greater than 480 s during cardiopulmonary bypass. After weaning the patient from cardiopulmonary bypass, complete heparin reversal is achieved. A loading dose of clopidogrel (600 mg) is administered three hours post-surgery, then starting on postoperative day 1, dual antiplatelet therapy is initiated. In this manner, our population exhibited a median drainage loss of 500 mL from chest tubes within 24 h, with only one (2.8%) instance of surgical resternotomy for bleeding. Similarly, these results are comparable to those of the study by Zivkovic et al. [20], in which the management of antiplatelet therapy differs slightly by utilizing a loading dose of clopidogrel of only 300 mg. Our findings reinforce those reported in a review by Zhang et al. [21], in which the authors observed that the overall death/stroke/myocardial infarction rate was higher in the staged group than in the synchronous group and that the synchronous group had better 30-day outcomes even if these data could not be statistically compared. Moreover, we conducted a follow-up analysis, reporting favorable survival rates at 1, 5, and 10 years, along with optimal freedom from repeat endovascular procedures for both coronary and carotid interventions over a span of up to 10 years. In conclusion, our results suggest that the synchronous execution of carotid artery stenting and coronary artery bypass grafting may be a viable option for patients with combined carotid and coronary artery disease. This approach is associated with low mortality, minimal risk of both neurological and hemorrhagic complications, and favorable long-term outcomes, warranting more frequent adoption for this patient population.

### Limitations

The primary limitations of this study are its retrospective design and small sample size, which may reduce the generalizability and robustness of the findings. Additionally, all data are derived from a single-center experience, which may introduce bias related to the specific practices, protocols, and patient populations at that institution, and the study did not include a comparative analysis with patients undergoing alternative treatment approaches, such as staged carotid artery stenting combined with coronary artery bypass surgery or surgical carotid endarterectomy. Another limitation is the absence of postoperative imaging to assess for subclinical events. As a result, we cannot exclude the possibility that small, asymptomatic lesions, which could have had clinical significance, may have occurred without detection. However, a longer-term follow-up would have strengthened the study by providing insights into the durability of the outcomes and the potential for delayed complications. Longer follow-up would also allow for a more comprehensive assessment of long-term neurological and cardiovascular events, as well as survival outcomes, which are critical for evaluating the overall benefits and risks of combined revascularization strategies in this patient cohort. 

## 5. Conclusions

Simultaneous CAS and CABG is a safe procedure, associated with low in-hospital mortality and morbidity and low incidence of neurological and hemorrhagic events. Our protocol, which includes maintaining the patient in an awake state for real-time neurocognitive assessment and employing brain protection techniques, minimizes the risk of perioperative stroke. Additionally, the administration of antiplatelet therapy on the day of surgery, three hours after the completion of the procedure, does not increase the risk of postoperative bleeding. The median/long-term outcomes are encouraging, demonstrating both favorable survival rates and a significant freedom from subsequent endovascular procedures. We believe these findings warrant further investigation, ideally in future studies that include control groups or multicenter collaborations to better establish the clinical benefits and safety profile of this combined treatment

## Figures and Tables

**Table 1:** Preoperative characteristics.

	N (%); Median (IQR)
Age, median (IQR)	72 (64–77)
Female sex, n (%)	8 (22.2)
Hypertension, n (%)	31 (86.1)
Diabetes, n (%)	18 (50.0)
Dyslipidemia, n (%)	23 (63.9)
Smoke, n (%)	18 (50.0)
COPD, n (%)	1 (2.8)
Preoperative Atrial Fibrillation, n (%)	1 (2.8)
Preoperative Pacemaker, n (%)	0 (0.0)
LVEF %, median (IQR)	59 (47–62)
Previous stroke, n (%)	4 (11.1)
Previous TIA, n (%)	2 (5.6)
Creatinine, mg/dL, median (IQR)	1.01 (0.87–1.14)
Previous Cardiac Surgery, n (%)	1 (2.8)
EuroSCORE logistic, median (IQR)	5.26 (3.73–8.91)
EuroSCORE II, median (IQR)	2.80 (2.06–3.58)
Preoperative IABP, n (%)	1 (2.8)

IQR, interquartile range; COPD: Chronic obstructive pulmonary disease; LVEF: Left Ventricle Ejection Fraction; TIA: Transient Ischemic Attack; IABP: Intra-Aortic Balloon Pump.

**Table 2:** Intra-operative characteristics.

	N (%); Median (IQR)
Stent type, n (%)	
• XACT	14 (38.9)
• Roadsaver	10 (27.8)
• Carotid Wallstent	12 (33.3)
Embolic protection	36 (100)
• Mo.Ma Ultra proximal Cerebral Protection	16 (44.4)
• Distal Filters	20 (65.6)
Off pump, n (%)	5 (13.9)
CPB time, median (IQR)	66 (54–89)
Cross-clmap time, median (IQR)	51 (41–72)
Associate procedures, n (%)	8 (22.2)
• Isolated Aortic valve Replacement, n (%)	3 (8.3)
• Isolated Mitral valve surgery, n (%)	4 (11.1)
• Combined Mitro-Aortic valve surgery, n (%)	1 (2.8)

IQR, interquartile range; CPB: Cardio-Pulmonary Bypass.

**Table 3:** In-hospital outcomes.

	N (%); Median (IQR)
Blood transfusions, n (%)	26 (72.2)
Post-operative MI, n (%)	2 (5.6)
Post-operative IABP, n (%)	1 (2.8)
cardiac_tamponade, n (%)	1 (2.8)
Post-operative stroke, n (%)	0 (0.0)
Post-operative TIA, n (%)	1 (2.8)
Bleeding requiring resternotomy, n (%)	1 (2.8)
Pericardiocentesis, n (%)	1 (2.8)
Acute kidney injury, n (%)	4 (11.1)
Postoperative dialysis, n (%)	1 (2.8)
Permanent Pacemaker implantation, n (%)	1 (2.8)
New onset atrial fibrillation, n (%)	9 (25.0)
Respiratory_failure (Re-intubation or intub > 48 h), n (%)	7 (19.4)
Pneumonia, n (%)	2 (5.6)
Wound complications, n (%)	0 (0.0)
In-hospital death, n (%)	0 (0.0)
Ventilation time, median (IQR)	9 (6–12)
Bleeding 24 h, median (IQR)	500 (325–700)
ICU stay, median (IQR)	2 (2–4)
In-Hospital stay, median (IQR)	8 (7–11)

IQR, interquartile range; MI: Myocardial Infarction; IABP: Intra-Aortic Balloon Pump; TIA: Transient Ischemic Attack; ICU: Intensive Care Unit.

## Data Availability

The data presented in this study are available on request from the corresponding author. The data are not publicly available due to data protection directive 95/46/EC.
